# Supplementary material for: Identification of Anoikis-Related Subgroups and Prognosis Model in Liver Hepatocellular Carcinoma
Source: Int J Mol Sci. 2023 Feb 2;24(3):2862. doi: 10.3390/ijms24032862 (PMC9918018; doi:10.3390/ijms24032862)
Supplement: Supplementary file 1 [file ijms-24-02862-s001.zip › Supplementary material.pdf]

Supplementary material

The number of Supplementary Figures: 7
The number of Supplementary Tables: 0

Supplementary Figure Legends

Supplementary Figure 1. Identification of anoikis-related subgroups.

(A). Uniform Manifold Approximation and Projection (UMAP) analysis of two groups. X-axis represents the UMAP1 and y-axis represents UMAP2.

(B). t-distributed Stochastic Neighbor Embedding (t-SNE) analysis of two groups. X-axis represents the tSNE1 and y-axis represents tSNE2.

Supplementary Figure 2. Identification of the highly correlated gene modules in WGCNA.

(A). The dendrogram of cluster in TCGA samples.

(B). Gene correlation scatter plot of the brown module. X-axis represents module Membership (MM), which is the correlation between genes and modules. Y-axis represents Gene Significance (GS), which is the correlation between genes and the clinical traits. p-value < 0.05

(C). Gene correlation scatter plot of the blue module. X-axis represents module Membership (MM), which is the correlation between genes and modules. Y-axis represents Gene Significance (GS), which is the correlation between genes and the clinical traits.

Supplementary Figure 3. GO-CC and GO-MF terms enrichment of genes in the highly correlated genes.

(A). GO-Cell Component (CC) terms enrichment of genes in the turquoise module. X-axis represents the number of genes in GO-CC term and y-axis represents enriched GO-CC terms. p-value < 0.05.

(B). GO-Molecular Function (MF) terms enrichment of genes in the turquoise module. X-axis represents the number of genes in GO-MF term and y-axis represents enriched GO-MF terms. p-value < 0.05.

Supplementary Figure 4. Construction of anoikis-related prognostic risk model.

(A). The time-dependent ROC curve of the performance of the prognostic model at 1, 3 and 5 years in training dataset. X-axis represents false positive rate and y-axis represents true positive rate. The AUC value represents area under the curve.

(B). The boxplot of expression of prognostic genes in different risk groups in training dataset. X-axis represents the five prognostic genes and y-axis represents the expression level of prognostic genes. Blue represents high risk group and orange represents low risk group.

(C-G). The overall survival of different risk groups of single gene including *BAK1* (C), *SPP1* (D), *BSG* (E), *PBK* (F) and *DAP3* (G) in training dataset. Blue represents patients in high risk group and red represents patients in low risk group.

****P < 0.0001.

Supplementary Figure 5. Validation of the prognostic model.

(A-E). The overall survival of different risk groups of single gene including *BAK1* (A), *BSG* (B), *SPP1* (C), *PBK* (D) and *DAP3* (E) in ICGC dataset. Blue represents patients in high risk group and red represents patients in low risk group.

(F). The heatmap of gene expression of prognostic genes in ICGC dataset. Blue represents low risk group and pink represents high risk group.

Supplementary Figure 6. Immunohistochemistry (IHC) validation of prognostic genes in tumor and normal samples in HPA database.

Supplementary Figure 7. Different characteristics of immune cell infiltration between two risk groups.
(A-H). Correlation of risk score with immune cells including resting dendritic cells (A), activated mast cells (B), gamma delta T cells (C), plasma cells (D), macrophage M0 (E), macrophage M2 (F), follicular helper T cells (G), resting mast cells (H).
