# Supplementary figures and images for: Identification of Anoikis-Related Subgroups and Prognosis Model in Liver Hepatocellular Carcinoma
Source: Int J Mol Sci. 2023 Feb 2;24(3):2862. doi: 10.3390/ijms24032862 (PMC9918018; doi:10.3390/ijms24032862)

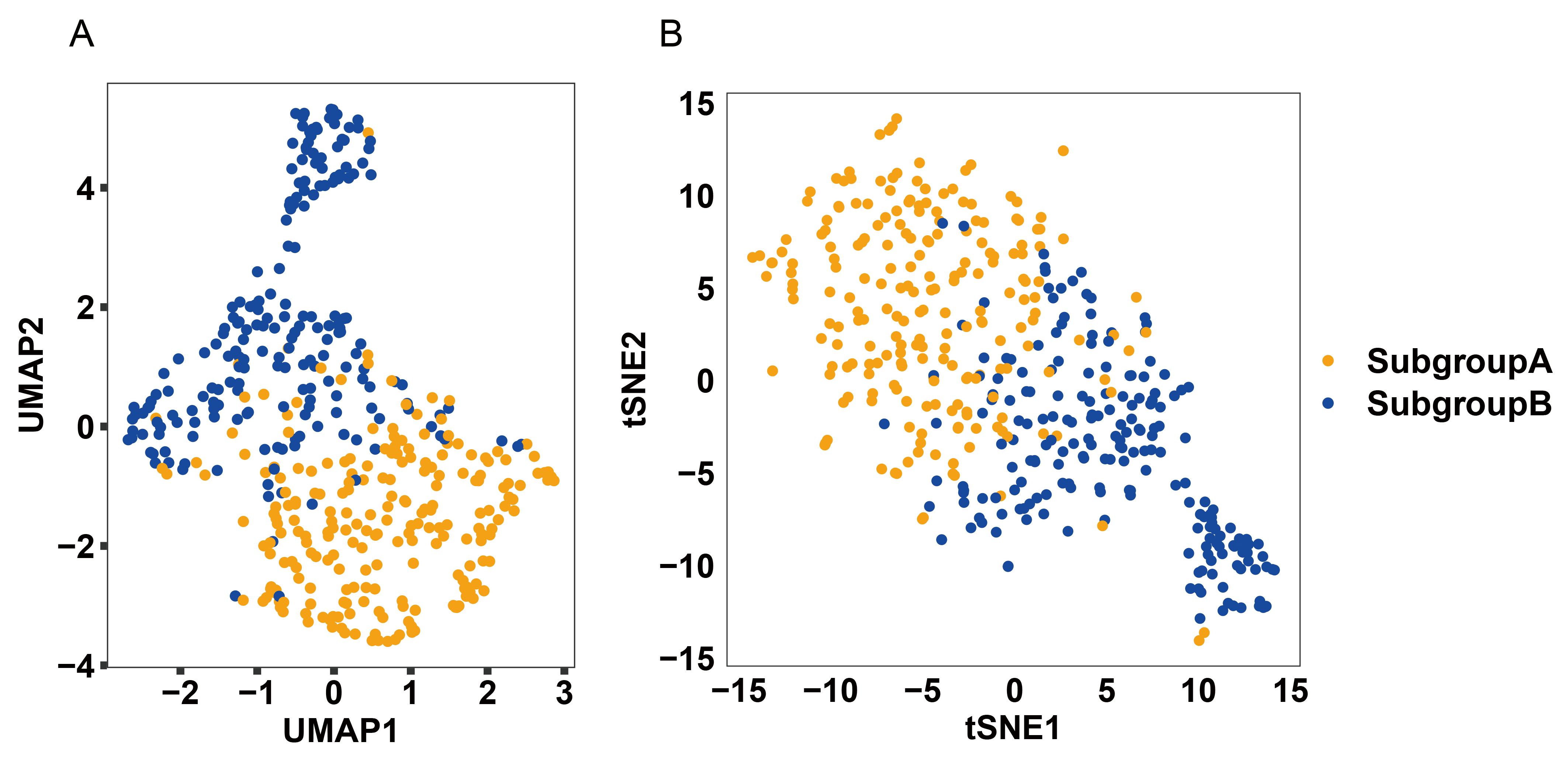

Supplement: Supplementary file 1 [file ijms-24-02862-s001.zip › Supplementary Figure 1.tif]

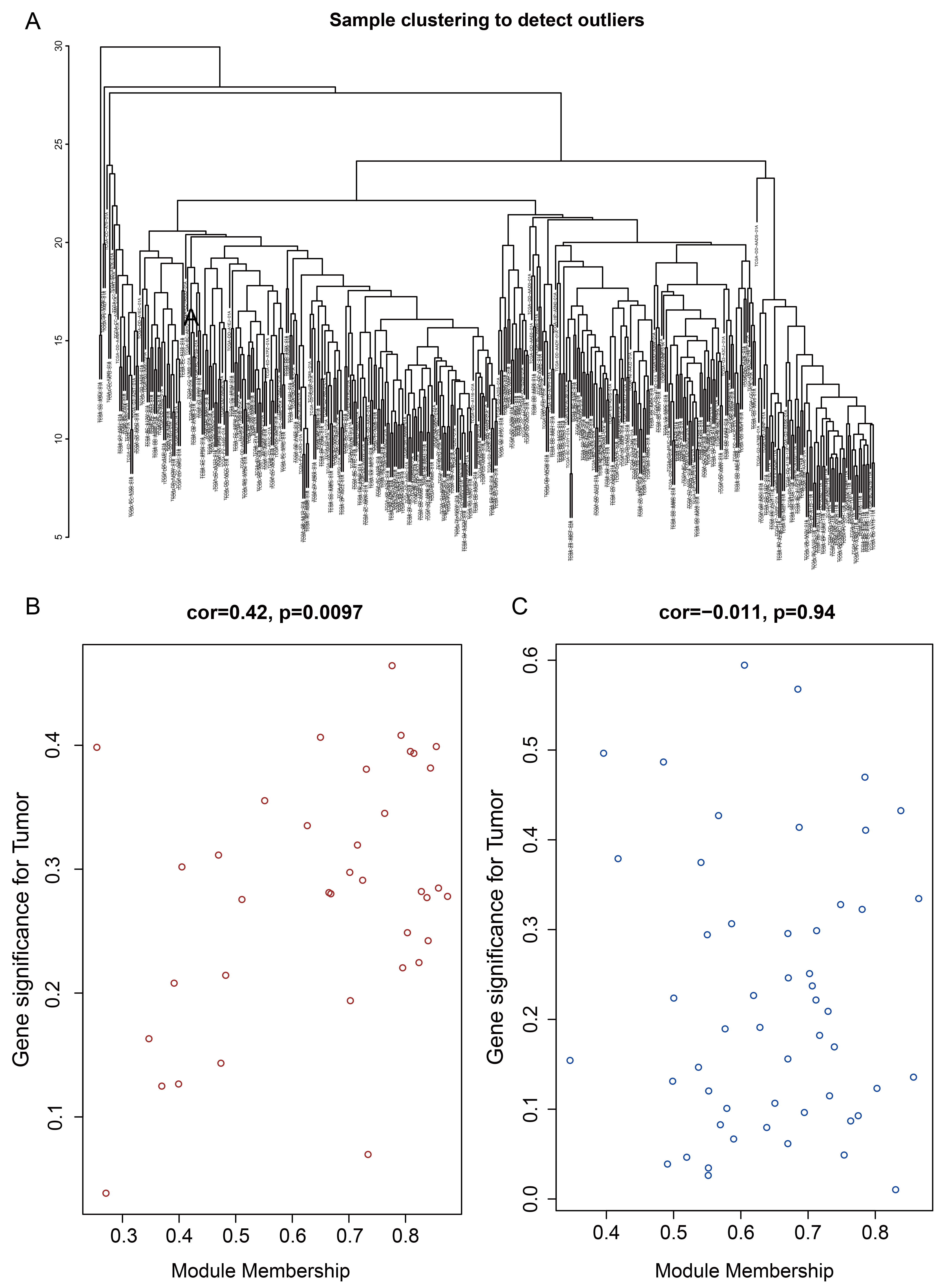

Supplement: Supplementary file 1 [file ijms-24-02862-s001.zip › Supplementary Figure 2.tif]

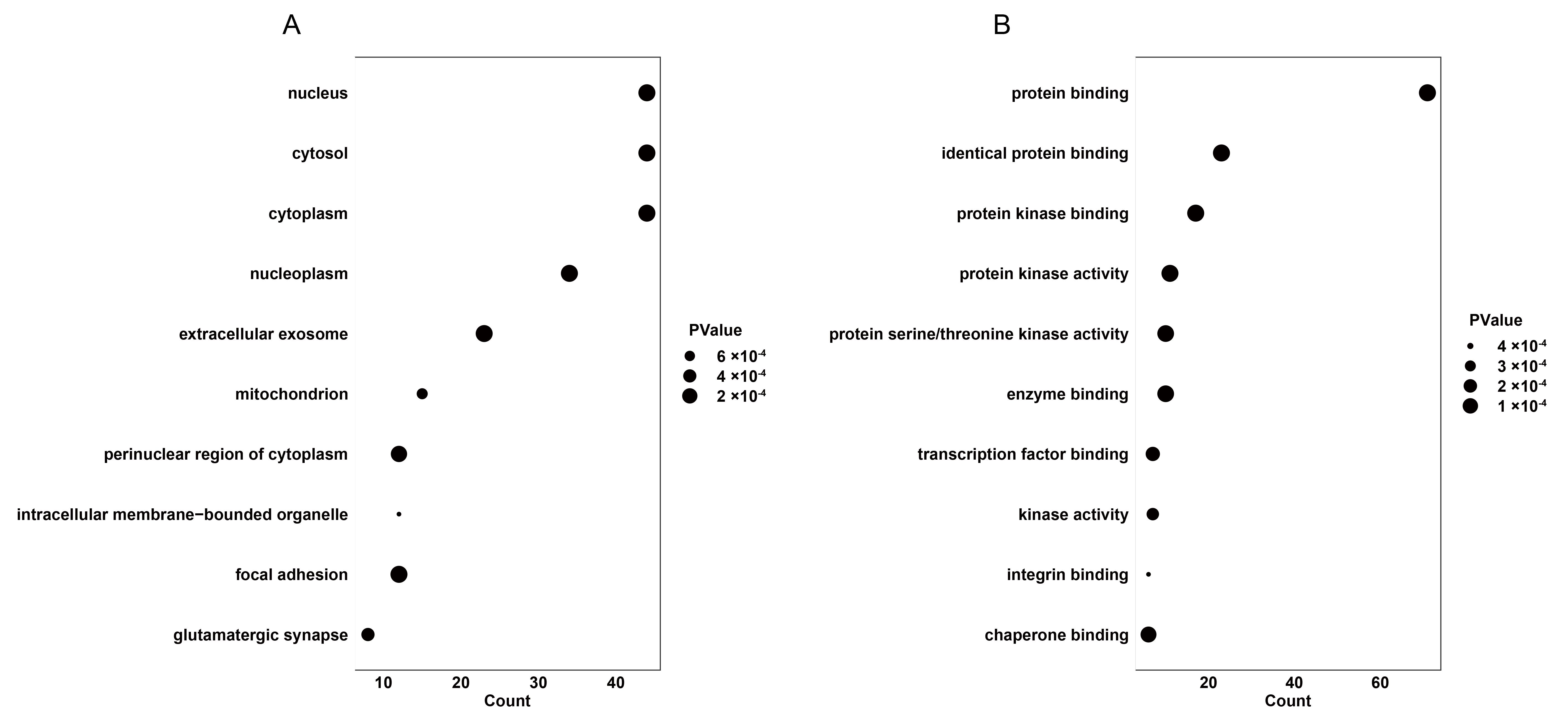

Supplement: Supplementary file 1 [file ijms-24-02862-s001.zip › Supplementary Figure 3.tif]

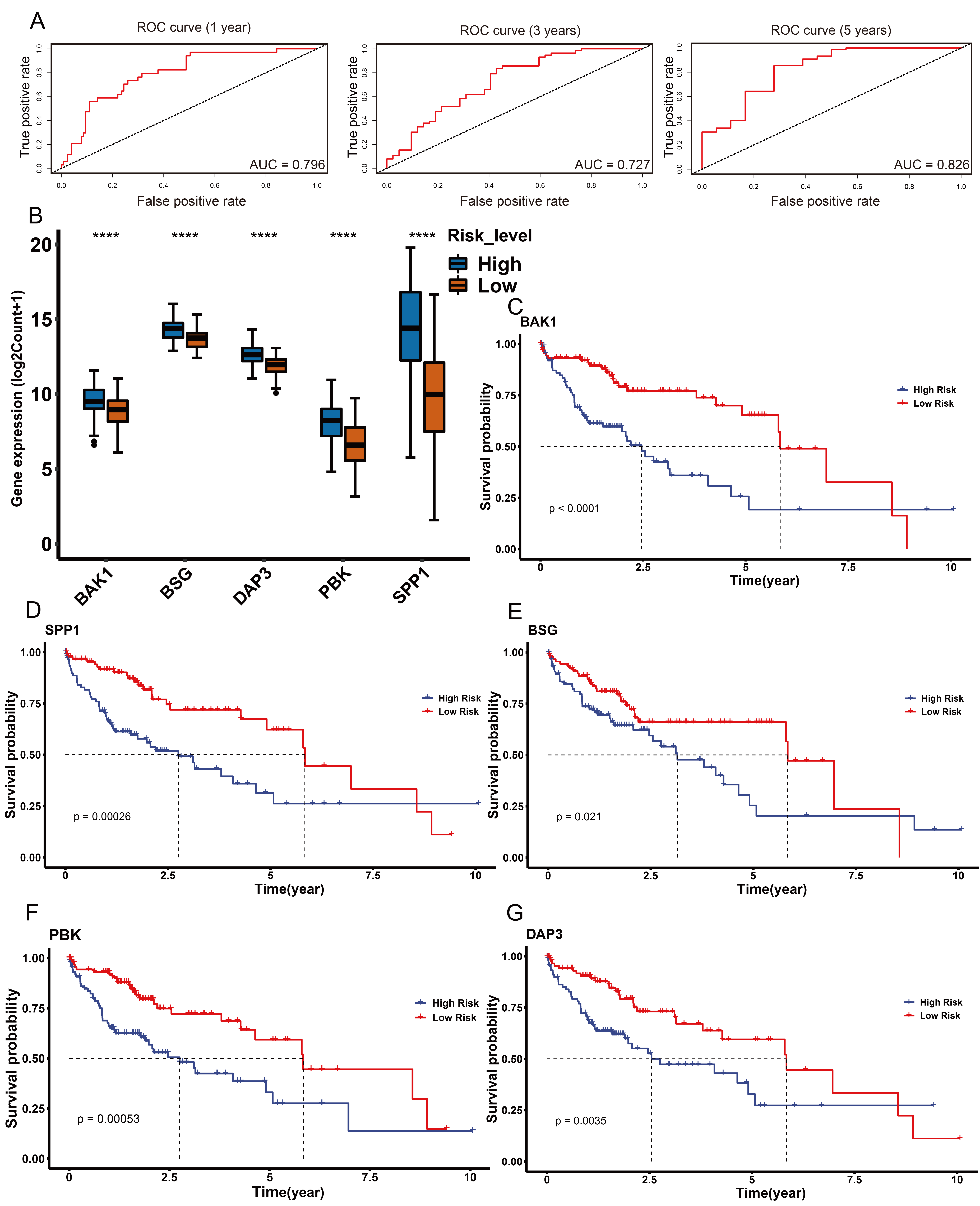

Supplement: Supplementary file 1 [file ijms-24-02862-s001.zip › Supplementary Figure 4.tif]

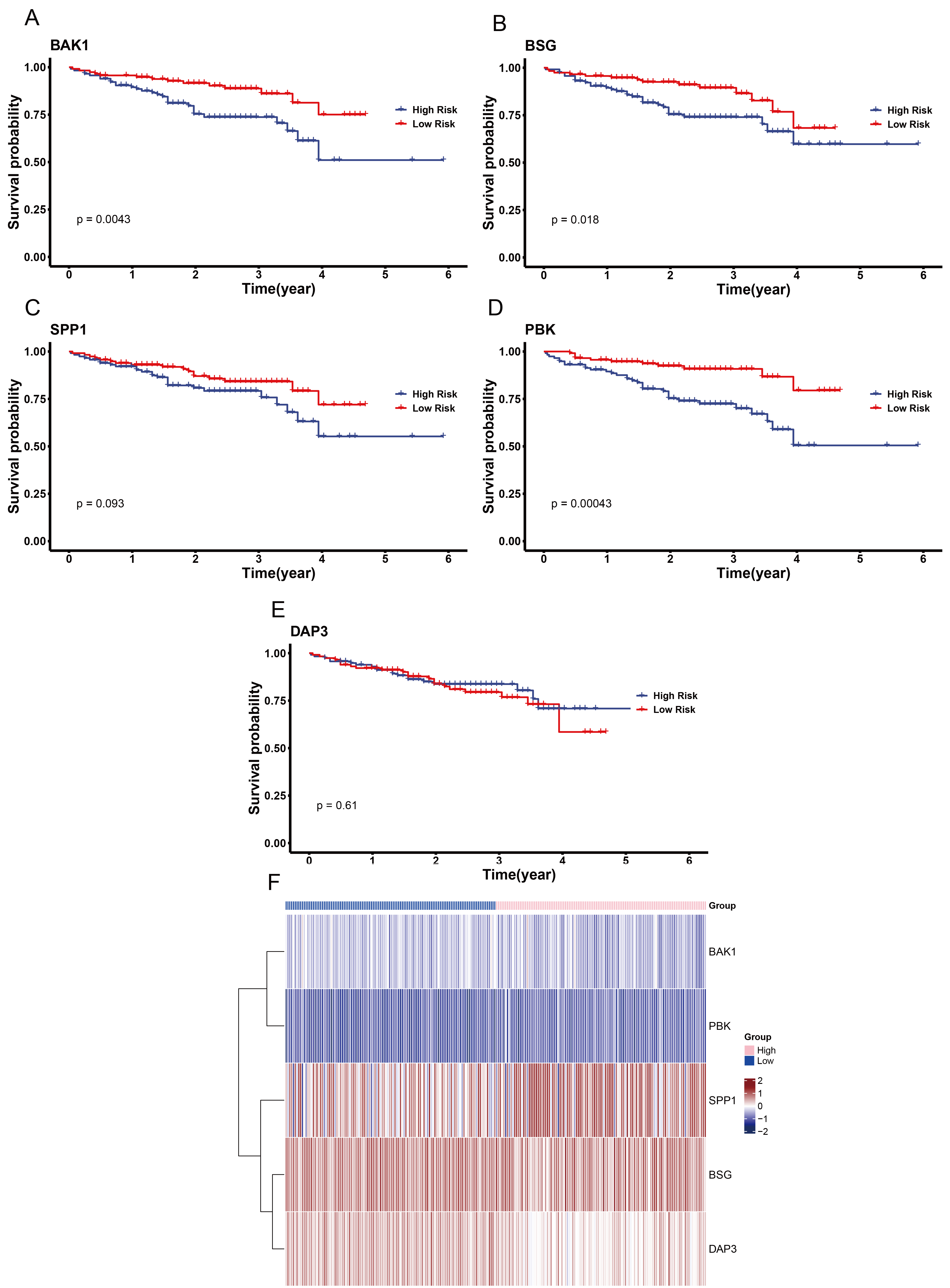

Supplement: Supplementary file 1 [file ijms-24-02862-s001.zip › Supplementary Figure 5.tif]

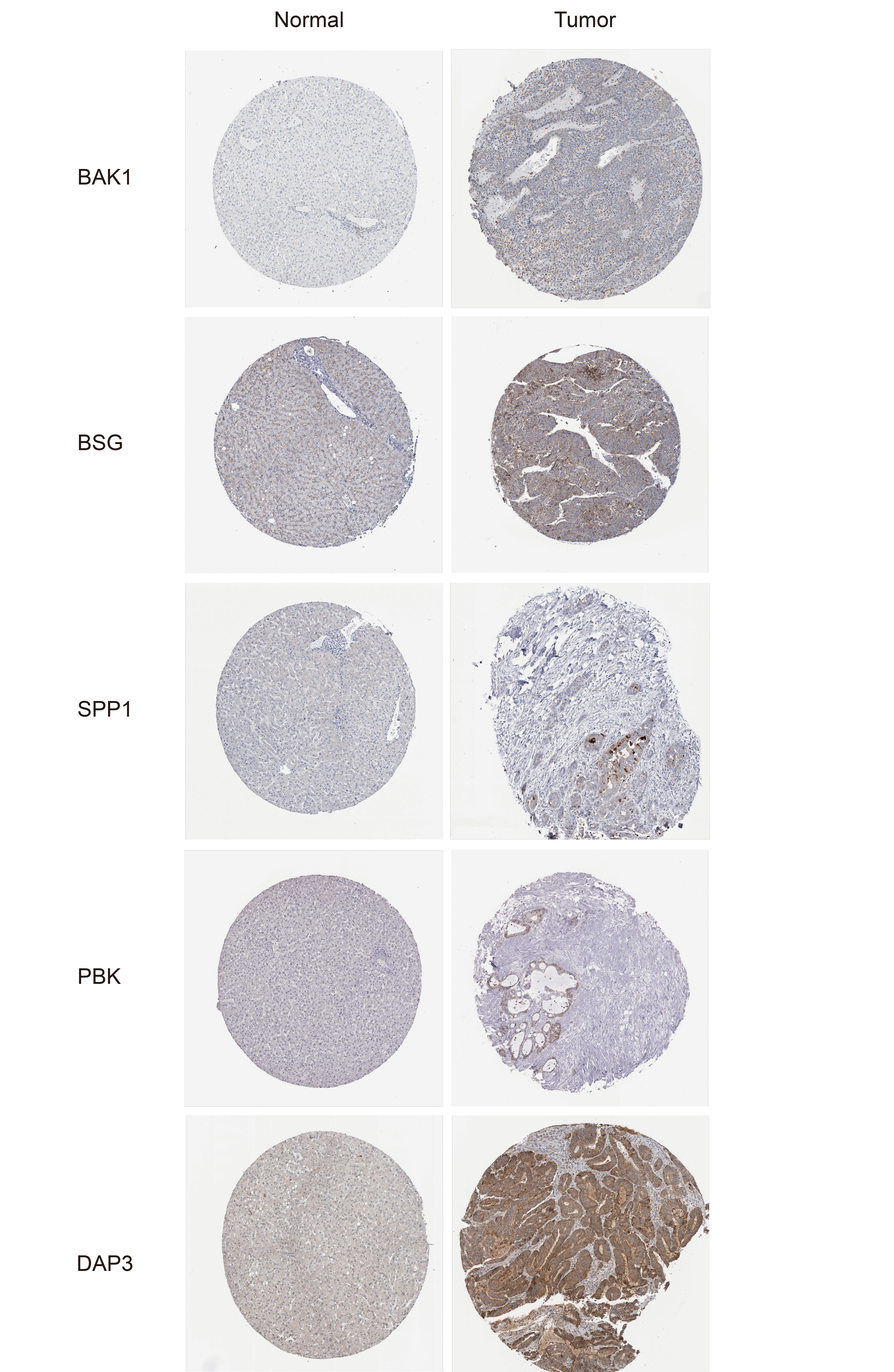

Supplement: Supplementary file 1 [file ijms-24-02862-s001.zip › Supplementary Figure 6.tif]

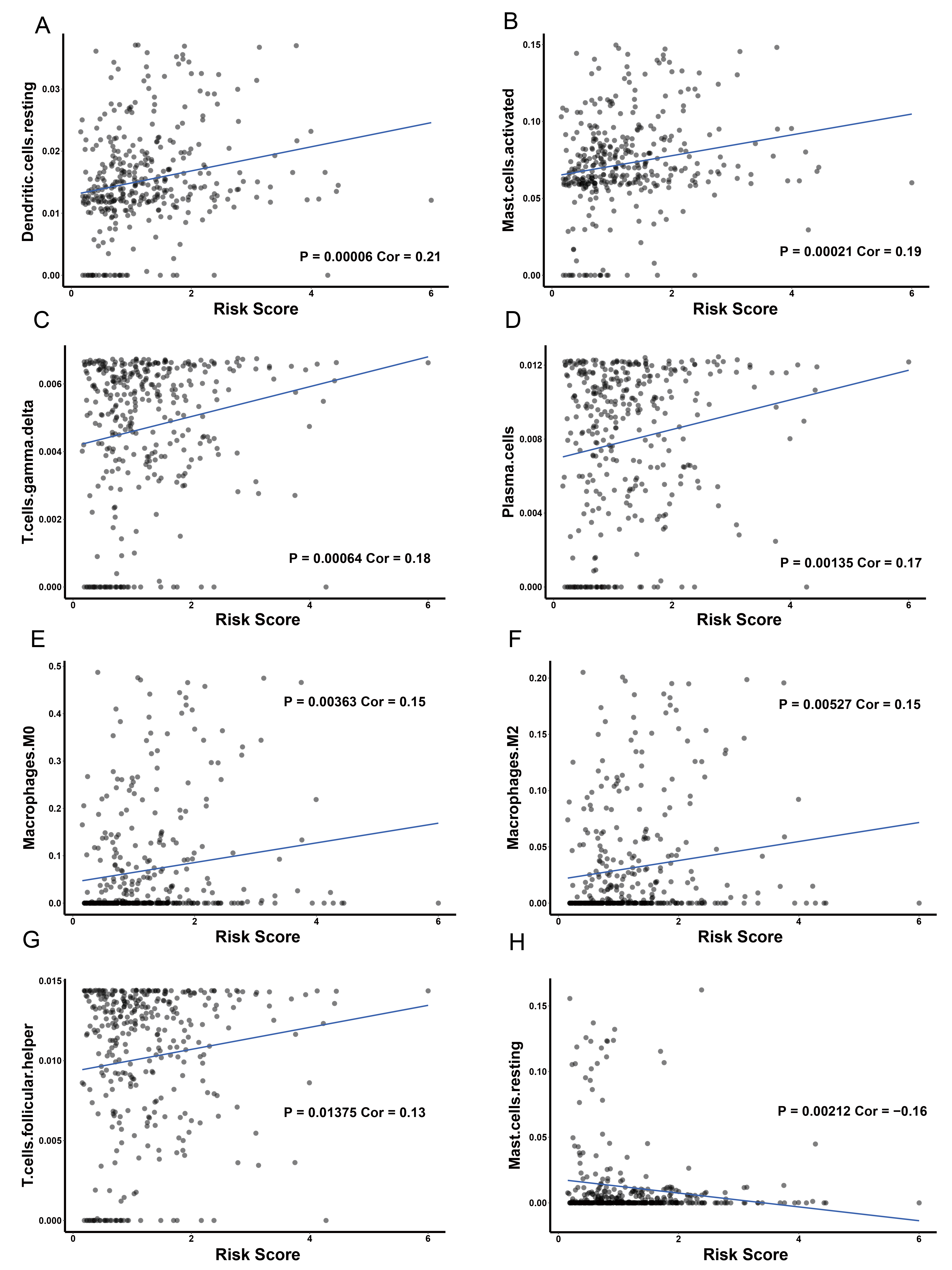

Supplement: Supplementary file 1 [file ijms-24-02862-s001.zip › Supplementary Figure 7.tif]
